# Immunoception: defining brain-regulated immunity

**DOI:** 10.1016/j.neuron.2022.10.016

**Published:** 2022-11-02

**Authors:** Tamar Koren, Asya Rolls

**Affiliations:** 1 Department of Immunology, Rappaport Faculty of Medicine, Technion - Israel Institute of Technology, Haifa, Israel; 2 Department of Neuroscience, Rappaport Faculty of Medicine, Technion - Israel Institute of Technology, Haifa, Israel

## Abstract

The emerging understanding of homeostatic neuroimmune interactions requires developing relevant terminology. In this NeuroView, Koren and Rolls define *immunoception* as the brain’s bidirectional monitoring and control of immunity. They propose that the physiological trace storing immune-related information, the *immunengram*, is distributed between the brain and memory cells residing in peripheral tissues.

In the current NeuroView, we describe a potential framework and terminology to encompass the increasing evidence of the brain’s sensing and regulation of peripheral immunity. We suggest that the brain receives inputs from the peripheral immune system to formulate a neuronal representation of the organism’s immunological state, a process that we define here as *immunoception*. We further define two types of programs that can be activated by the nervous system to regulate immunity, inborn and acquired. The *inborn neuro-immune responses* are based on an innate set of reactions, shaped by collective evolutionary constraints. Conversely, *acquired neuro-immune responses* are generated by each organism forming its own unique reactions based on experience. Finally, we propose that the physiological memory trace representing a past immune experience, the *immunengram*, is distributed between a central neuronal representation, and the resident cells located in the peripheral tissues.

## Immunoception

The nervous system monitors the organism’s internal state on an ongoing basis, a concept known as interoception. Interoception encompasses inputs regarding metabolism, temperature, and visceral sensations, as well as internal signals such as pain, thirst, hunger, and potentially, immunity (Chen et al., 2021). Given that the immune system continuously patrols the body to eliminate pathogens and detect changes in tissue composition and distress (Kipnis, 2018), it carries information that is crucial for the brain’s ability to evaluate the organism’s state and its integrity.

Indeed, the brain responds to changes in peripheral immunity (Kraynak et al., 2018). Yet, the aspects of the immune process that the brain recognizes, and to which of these it responds, are still subject to debate. We know that the brain is sensitive to changes in the intensity of inflammation, as arthritis patients treated with an anti-TNFα drug demonstrated changes in brain activity even before clinical improvement occurred (Hess et al., 2011). The brain can be potentially informed about the type of an invading pathogen, as vagal afferents and other sensory neurons express receptors, such as Toll-like receptors (TLRs), recognizing pathogen-related components (Pavlov and Tracey, 2017; Pinho-Ribeiro et al., 2018). In addition, it has been shown that the anatomical location of a given inflammation induces a differential c-Fos expression pattern in hypothalamic paraventricular nucleus (PVN) neurons (Belevych et al., 2010).

Reciprocally, the brain can initiate corrective programs to regulate the immune response. For example, sepsis and an overwhelming immune activation, which can lead to major damage to the organism, is accompanied by brain-induced changes in feeding behavior and metabolism (Wang et al., 2016). Moreover, sepsis also leads to the activation of a corrective response, the “inflammatory reflex”, an anti-inflammatory parasympathetic reaction (Pavlov and Tracey, 2017).

Thus, as a result of immunoception, the brain can generate a prediction suggesting the appropriate corrective response, or action plan, required to restore homeostasis. If the induced corrective response is effective, the prediction is validated and does not require an update of the immune representation in the brain. However, if the induced action plan does not lead to the anticipated outcome, the neuro-immune scheme must be updated. The new/modified representation should be integrated into the existing neuro-immune scheme to generate an updated image in the nervous system of the peripheral immune state, as part of the ongoing process of immunoception.

## Inborn vs acquired neuro-immune responses

The nature of the induced corrective neuro-immune response can be either inborn or acquired based on experience. This is in analogy to the concept of innate vs acquired responses in the immune system, and the instinctive vs learning and memory processes in neuroscience.

*The inborn neuro-immune response*, as proposed here, represents an action plan that is based on a set of evolutionary adaptations reflecting the collective experiences of the species. Thus, some evolutionarily conserved neuronal functions predict an upcoming potential immune challenge and initiate a set of typical immunological responses. For example, mating can expose an animal to pathogens by horizontal social transmission, thus potentially benefiting from increased immune protection. Such social interactions are known to activate defined brain regions linked to the reward system. Accordingly, we showed that activation of the ventral tegmental area (VTA), a key component of the reward system, boosts anti-bacterial immunity (Ben-Shaanan et al., 2016). This concept was directly demonstrated in the phenomenon of mating anticipation, in which male mice that encounter females were shown to induce a typical increase in serum IL-2 (Kayama et al., 2022). This prototypic increase in cytokines was shown to be dependent on reward system activity, as it was attenuated by inhibiting the VTA (Kayama et al., 2022). As another example, hypothalamic corticotrophin-releasing hormone (CRH) neurons, which orchestrate behavioral and endocrine responses to stress, enhance mobilization of peripheral immune cells between compartments, thereby altering the acquisition of adaptive immunity to viral infections and self-antigens (Poller et al., 2022). Sleep and circadian rhythms, centrally regulated by the hypothalamic suprachiasmatic nuclei, have been shown to affect cytokine production by myeloid cells in the peripheral blood (Besedovsky et al., 2012). Thus, these examples of inborn neuro-immune responses represent conserved connections between triggers, a specific neuronal activity, and the relevant immune outcome.

*The acquired neuro-immune response* is formed based on an experience that generates a novel association between certain neuronal activity and a specific immune process. The best example of such interactions is immune conditioning, in which a specific immune response (e.g., immune suppression) is associated with a non-immunogenic stimulus (e.g., a certain tastant). Such adaptive neuro-immune interactions are relevant, for example, if an organism occasionally feeds on a certain food source that is contaminated with a pathogen. Anticipating exposure to the pathogen can be beneficial as it enables the individual to initiate the relevant immune response even before exposure to the antigen actually occurs. Immune conditioning has been shown to be dependent on specific brain regions, such as the amygdala and insular cortex (Hadamitzky et al., 2020), reinforcing the notion that these regions are involved in the learning process of conditioned immune responses.

## The immunengram

The term engram, depicting a memory trace, encompasses the neural substrate underlying the storage and recall of memories (Josselyn and Tonegawa, 2020). Thus, it has been established that memories are encoded in specific neuronal ensembles, and can be recalled by reactivation of the same groups of neurons that were initially activated during the original encoded experience (e.g., in fear conditioning). Similarly, it is possible that throughout its constant monitoring, once the brain encounters an immunological event that deviates from the expected, relative to previously encoded patterns (e.g., a newly-encountered pathogen or vast tissue damage), the brain forms a new or adapted neuronal trace (see acquired neuro-immune responses), namely, an immunengram.

Recently, our group has demonstrated that previous immune experiences can be retrieved by the reactivation of specific neuronal ensembles in the insular cortex that were active during the original inflammatory episode (Koren et al., 2021). This retrieval was not apparent following general activation of the insula; it manifested anatomical specificity, and could recapitulate the specific immune response observed during the original immune experience. These findings suggest that some aspects of brain-immune regulation are dependent on a prior experience, showing memory-like (i.e., mnemonic) features.

In our study (Koren et al., 2021), we focused on the insular cortex, yet it is important to note that the insula is likely only a part of the neuronal network in the brain that is involved in peripheral immune processing and modulation. Additional brain regions, such as the hypothalamus, thalamus, amygdala, somatosensory cortex, and anterior cingulate cortex, demonstrate increased neuronal activity during a heightened inflammatory response (Kraynak et al., 2018), rendering these areas potential contributors to a cross-regional immunengram.

Notwithstanding, we suggest that in the immunengram representation, in deviation from the classical concept of an engram, the neuronal trace is necessary, but it is not sufficient. While the brain and the immune system can both encode complex information, the pathways mediating information transfer, the efferent neurons of the autonomic nervous system and the endocrine mediators, have only a limited set of potential responses. This raises an enigma regarding how the brain can deliver complex inputs to the immune system. Thus, we suggest that the term immunengram represents not only a neuronal memory trace but also a trace dispersed in parallel in the previously inflamed tissue in the form of changes in the tissue cells (e.g., increased expression of neuropeptide and neurotransmitter receptors) and specific immune clones (e.g., memory lymphocytes) that remain in the tissue after the inflammation. Thus, upon reactivation of neurons comprising the neuronal component of the engram, the downstream peripheral pathways can activate these tissue-resident cells. These tissue components “interpret”, based on their own information (e.g., antigen-specific clones), the relatively limited peripheral neuronal input and eventually recapitulate part of the complexity of the tissue’s previous inflammatory event.

**Figure 1:**
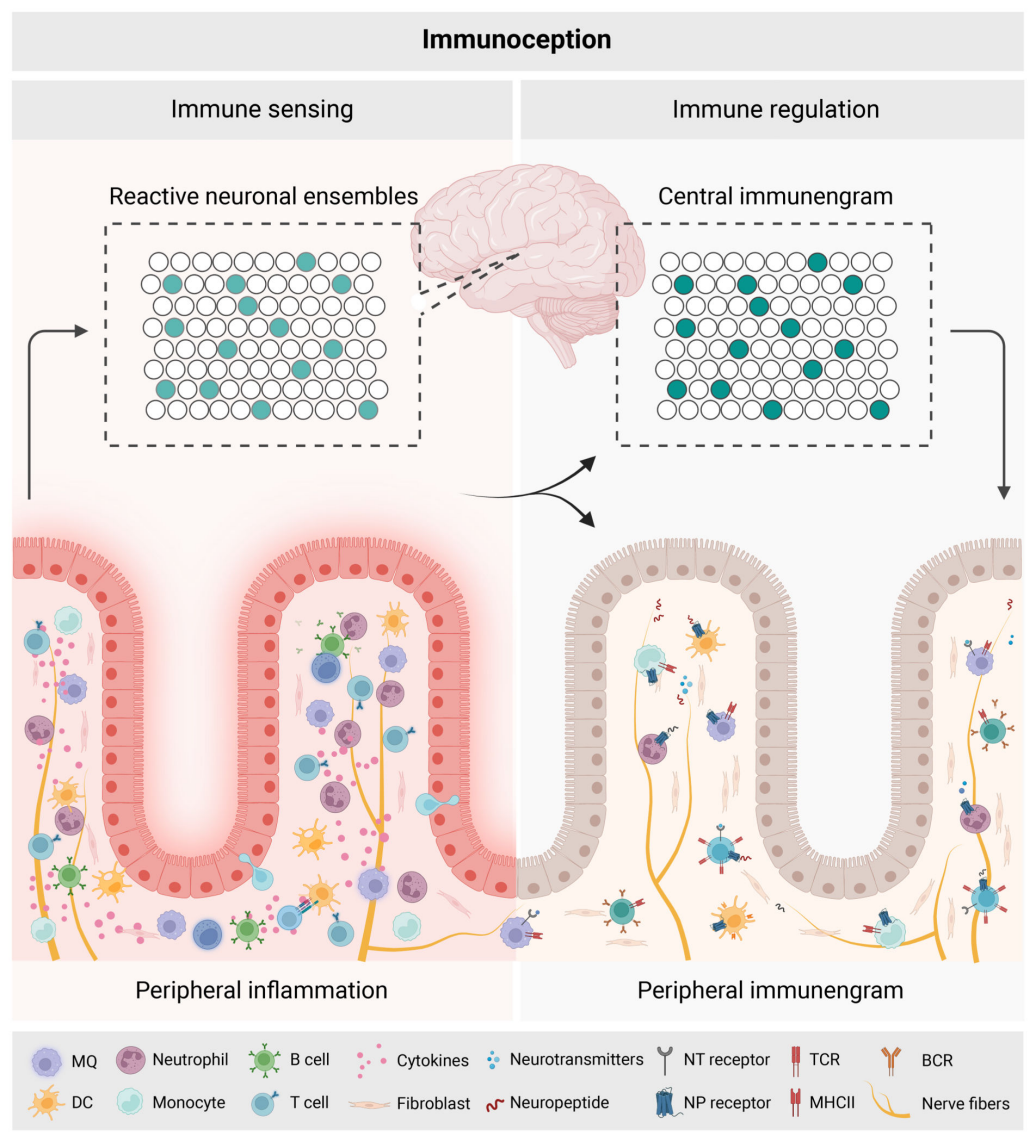
Schematic model of immunoception and the distributed immunengram. During peripheral inflammation, specific neuronal ensembles in the brain are activated and encode immune-related information. Upon resolution, the physiological trace representing the immune experience, the immunengram, is distributed between a central neuronal representation, and cells that remain in the peripheral tissues. The latter includes the presence of antigen-specific memory immune cells (e.g., T and B cells and ILCs) and increased sensitivity to central-derived local neuronal inputs (via expression of neuropeptide- and neurotransmitter-receptors on resident cells). ILCs, innate lymphoid cells; MQ, macrophage; DC, dendritic cell; NT, Neurotransmitter; NP, neuropeptide; TCR, T cell receptor; BCR, B cell receptor. Created with BioRender.com.

Taken together, we suggest that retrieval by the brain of a distinct immune response, with anatomical and immunotypic specificity, requires two conditions: First, a specific neuronal ensemble in the brain that encodes the immune-related information; and second, immune and tissue cells at the peripheral site of inflammation that became sensitive to the local innervation and are ready to act upon their reactivation (Figure 1).

## Summary

In this NeuroView, we suggest that the brain continuously monitors the peripheral immune state, in a process that we named immunoception. Based on information from the immune system, the brain forms a representation of the immune state, and can initiate a corrective neuro-immune reaction. This reaction can be inborn, sculpted by the evolutionary experiences of the species, or acquired, based on one’s own experience. To represent the acquired responses and their accompanying associations, we use the term immunengram to describe a distributed memory trace between the brain and the peripheral tissue.

The proposed terminology integrates and formulates the emerging evidence for the central physiological role of neuroimmune interactions in health and disease. Yet, these concepts also highlight the major gaps that remain in the field, such as how does the brain integrate interoceptive, pain and immune responses? What are the neuronal components of the immunengram, where is it encoded in the brain, and what are the peripheral components of this representation? If the immune representation forms the basis for predictions, what is the mechanism for generating these predictions and how is their outcome evaluated? Addressing these and other questions in the field will develop our understanding of the neuroimmune interactions. Concomitantly, it will naturally modify the proposed framework, to accommodate newly discovered physiological mechanisms underlying the brain-body connection.
